# 57-Year-Old Male Veteran with Recurrent Fevers

**DOI:** 10.5811/cpcem.1546

**Published:** 2023-08-09

**Authors:** Eileen Chu, Samantha A. King, Laura J. Bontempo, T. Andrew Windsor

**Affiliations:** * University of Maryland Medical Center, Baltimore, Maryland; † University of Maryland School of Medicine, Department of Emergency Medicine, Baltimore, Maryland

## Abstract

A 57-year-old male veteran presented to the emergency department for recurrent fevers for 10 days. The patient was febrile but had an overall benign physical exam. This interesting case explores the broad differential diagnosis and evaluation in a patient who presents with fever of unknown origin.

## CASE PRESENTATION (Dr. Chu)

A 57-year-old male veteran presented to the emergency department (ED) with recurrent fevers. The patient reported that he’d had a fever for 10 nights with a maximum temperature of 104° Fahrenheit (F)/40° Celsius (C). He also mentioned having right upper quadrant (RUQ) abdominal pain for about a week, which he believed was related to his inflammatory bowel disease (IBD). He stated that he went to an urgent care on three separate occasions for his symptoms where he tested negative multiple times for severe acute respiratory syndrome coronavirus 2 (SARS-CoV-2) and influenza. The urgent care obtained laboratory studies, which showed a mild leukocytosis of 14,000 white blood cells per microliter (mcL) on the second visit and 12,000/mcL on the third visit. On one of his visits, the patient complained of a headache and reported prior sinus infections; so he was started on doxycycline for presumed sinusitis. When asked about new medications, the patient reported the initiation of infliximab infusions for his IBD two months earlier and was last infused the month prior to the ED visit. He denied any recent foreign travel or exotic animal exposures.

The patient’s past medical history included IBD, hyperlipidemia, and obstructive sleep apnea. He had no past surgical history. His social history included prior heavy alcohol use (although he reported quitting in 2015), and no tobacco or illicit drug use. His home medications were atorvastatin nightly, diclofenac as needed, lactase as needed, and infliximab monthly. He had no known drug allergies. Greater than 10 years ago, the patient reported being stationed in areas such as Japan, Bahrain, Qatar, Egypt, Kuwait, and Cyprus. His review of systems was positive for abdominal pain, a single episode of diarrhea, myalgias, chronic back pain, and a headache. He denied any upper respiratory symptoms, such as cough, sore throat, or rhinorrhea. He denied any chest pain, shortness of breath, nausea, or vomiting.

On examination, the patient was in no acute distress, but he appeared uncomfortable. He was febrile to 101.8°F/38.8°C with a heart rate of 94 beats per minute, blood pressure 154/77 millimeters of mercury, and his oxygen saturation was 95% on room air. He weighed 98.4 kilograms (body mass index = 32.0 kg/m^2^). His head was normocephalic and atraumatic. His sclera were anicteric and his pupils were equal, round, and reactive to light. His oropharynx was clear and moist. His neck was supple with normal range of motion and without meningeal signs. His heart had a regular rate and rhythm without any murmurs, rubs or gallops. His lungs were clear to auscultation bilaterally without wheezes, rales, or rhonchi. Despite the patient’s complaint of RUQ pain, his abdomen was soft, nondistended, and nontender. There was no hepatomegaly. His skin examination was without any rash. He had full range of motion of all extremities. His neurologic exam was without gross abnormalities.

**Image 1:**
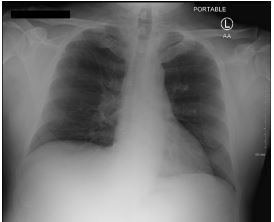
Portable chest radiograph of a 57-year-old male veteran with recurrent fevers.

The patient was given one gram of acetaminophen and received one liter of normal saline intravenously (IV). Laboratory studies, a respiratory viral panel swab, and blood cultures were obtained. A chest radiograph was performed (Image 1). The patient’s initial lab results are shown in the Table. His labs were significant for a mild leukocytosis. His chemistry panel showed a decreased bicarbonate level, anion gap of 15, and very mildly elevated liver function tests. He had a normal lactate and urinalysis (UA). His coagulation studies were elevated.

In the ED, an additional test was ordered, and a diagnosis was made.

## CASE DISCUSSION (Dr. King)

This is a case of a 57-year-old male who presented to the ED with a chief complaint of intermittent fevers in the setting of an underlying autoimmune disorder, IBD. He had some other varying complaints in his history and review of systems including RUQ abdominal pain, which was thought to be attributed to his IBD, chronic back pain, and myalgias, headaches, and chills, which are all incredibly non-specific. He has a remote history of alcohol use disorder but was not actively drinking or using illicit substances. The team astutely obtained a detailed travel history, which noted that the patient has been out of the country on several occasions but not recently. Additionally, he was undergoing monthly infliximab infusions. Outside these key features, there is not much that is narrowed down from this history.

In the ED, his vitals were notable for a fever but were otherwise unremarkable. Additionally, despite the other complaints in his history, his abdominal examination was overall unremarkable with no complaints of tenderness on examination. The remainder of his examination was otherwise non-specific, similar to his history.

The team initiated a limited laboratory work-up looking for possible sources of the patient’s fever including a SARS-CoV-2 test (although he had previously tested negative), UA, and blood counts. There were a few key findings including very mildly elevated liver enzymes and international normalized ratio, without an elevated bilirubin. He also had a slight acidosis with a normal lactate and normal anion gap. His chest radiograph was unremarkable, without signs of infiltrates, consolidations, or effusions. Unfortunately, in combination with his relatively unremarkable history and physical, his lab work and imaging thus far are non-conclusive, and the differential diagnosis remains broad.

Creating a differential diagnosis for fever without an organized system can be overwhelming, risking having diagnoses be overlooked or missed. One system that can be applied here is to create a differential diagnosis for fever of unknown origin (FUO). While this patient does not technically meet the definition of FUO (which requires fever documented >38.3°C on several occasions over the course of three weeks with a one-week hospital work-up), systems for evaluating FUO can still be helpful as an organizational method for approaching patients who do not have an obvious underlying source of their fever.^1^ One such system uses the mnemonic I-MADE to organize the differential diagnosis.^2^ This mnemonic stands for infections, malignancy, autoimmune disorders, drug-induced, and everything else.

Within this mnemonic, infection is the broadest category and should be addressed last, unless there is a very high suspicion. The next category to consider is malignancy. This patient did complain of abdominal pain and had mildly elevated liver enzymes, which may suggest a primary or metastatic lesion to the liver. He was on the younger end of the age spectrum where malignancy would be expected but not out of the realm of possibility. However, the patient lacked other symptoms classically associated with malignancy such as weight loss or night sweats. Also, the time course would have been very rapid to have developed malignancy-related fevers over one to two weeks without any other symptoms. Therefore, malignancy is unlikely to be the cause of this patient’s presentation.

Next for consideration is autoimmune diseases. This patient already had an underlying autoimmune disease, and while it is possible that his IBD was the underlying cause of his symptoms, he was actively undergoing therapeutic treatment and one would expect his symptoms to be improving and not getting worse. Autoimmune disorders often come in clusters; so, it is possible that he developed an additional disorder. His infliximab treatment, however, would likely have been therapeutic for additional autoimmune disease, such as rheumatoid arthritis, that would lead to a fever,. He was also missing hallmark features of these diseases such as joint pain, rashes, or focal muscular tenderness/weakness. Thus, an additional autoimmune disorder was also unlikely to be the underlying cause.

The next category, drug-induced fevers, is difficult as it is only diagnosed by elimination since there does not typically exist a test for diagnosis. However, it is an important category to keep in mind. The patient was taking atorvastatin, a non-steroidal anti-inflammatory, and an over-the-counter lactose intolerance medication daily. None of these medications are likely to cause a fever, but the patient was additionally on infliximab infusions. Infliximab is known to cause many of the patient’s symptoms. Infliximab is associated with fevers, headaches, and liver enzyme elevation; all of which were key features of this case.^3^ While this diagnosis initially appears promising, it should only be the diagnosis of choice after all others have been eliminated. Additionally, his last infusion was nearly a month prior to presentation, and one would have expected to see symptoms sooner than this presentation.

The last category in this mnemonic is a catch-all of everything else. However, key diagnostic groupings to consider within this category include thromboembolism, endocrine disorders, neurologic dysregulation, environmental exposure, and factitious disorder. The patient did not present with any signs of thromboembolism such as shortness of breath, tachypnea, hypoxia, leg swelling or pain, so this is less likely to be the cause. For endocrinopathies and neurologic dysregulation, one would have expected to see other associated features such as skin changes, vital sign abnormalities, and/or lab abnormalities. Since these features were not present, these are less likely to be the cause. For the last two groupings of environmental exposure and factitious disorders, it is less likely that the patient would have a persistent fever once removed from the environmental source, and one should not assume a factitious disorder without ruling out of all other medical conditions.

This brings me back to the first category of infection. This category is broad and has several ways to further break it down, such as by body system or by type of infection (viral, fungal, etc). For body systems, the only focal areas concerning for infections were his abdomen (RUQ pain) and head (headaches). This would add an intra-abdominal abscess - most likely liver but sparing the biliary system given his normal bilirubin - and meningitis/encephalitis to the differential diagnosis. For the latter diagnoses, the patient was immunosuppressed but did not have typical associated signs or symptoms such as neck pain, nuchal rigidity, or altered mental status, and his headache was only noted on review of systems. In terms of a liver abscess, the patient had only mildly elevated transaminases and did not have tenderness on his examination. However, given his immunosuppressed status, it is possible once again that these might not have been as prominent. Unlike his headache though, his abdominal pain was more noteworthy and a focus of his presentation. Therefore, liver abscess remains on the differential.

Within the category of systemic infections, viral infections such as cytomegalovirus, human immunodeficiency virus, hepatitides, and Epstein-Barr virus are all possible. However, most of these viruses should have been screened for prior to initiating infliximab treatment or would have had other features such as a rash or changes to blood counts. Pathologies like parasitic and fungal infections, such as anaplasmosis, ehrlichiosis, or leptospirosis, often have anemia or, if invading the liver (to explain the RUQ pain), would have had a cholestatic pattern on lab testing. These infections are difficult to rule out but do not greatly explain the patient’s symptoms and predominantly normal lab work, including a non-elevated bilirubin and alkaline phosphatase. In a patient with international travel, malaria, dengue fever, and typhoid fever should be considered. Once again, the patient lacked key features for these diagnoses including anemia, rashes, and profuse diarrhea. Another consideration in patients with international travel is tuberculosis (TB). The patient should have been screened out for this disease prior to initiating infliximab; however, there are several reports of false-negative screening and presentation of TB after infliximab infusion. Therefore, TB must remain on the differential diagnosis.

While the list of possible infections can go on and on, focusing on the patient’s chief complaints along with the fever, narrows the differential diagnosis to liver abscess and TB. Ultimately, while the patient did not have RUQ tenderness on exam, this was a key history point, and he had the elevated liver enzymes on his lab work. While TB could explain these findings, case reports of occurrences after initial screening are rare, and it is likely, given the patient’s military history, that he would have undergone multiple screenings for TB in his lifetime.

Therefore, my final diagnosis is liver abscess, and my diagnostic test of choice would be computed tomography (CT) of the abdomen and pelvis with IV contrast. Of note, an ultrasound of this area would also be an appropriate initial test but would potentially miss other sources of abscess in this area; so in the undifferentiated patient, a CT would be the most preferred testing modality.

## CASE OUTCOME (Dr. Chu)

**Image 2:**
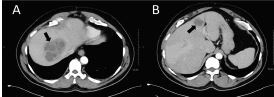
Computed tomography of the abdomen and pelvis with intravenous contrast of a 57-year-old male veteran with recurrent fevers identifying hypoenhancing lesions, which are consistent with a 9.2-centimeter (cm) abscess in the right hepatic dome (A) and a 3.5 cm abscess in hepatic segment 4 (B) (black arrows).

The diagnostic test ordered was a CT abdomen/pelvis with IV contrast (Image 2). The CT was interpretated by the radiologist as having two hypoenhancing lesions within the right hepatic dome, and these “findings could be compatible with hepatic pyogenic abscesses or hypoenhancing liver metastases, which could be differentiated based on clinical context.” The patient reconfirmed with us that he had no cancer history, recent international travel, or possible exotic exposures. Given the patient’s recurrent fevers, abdominal pain, recent infliximab infusion, and lack of cancer history or other symptoms such as fatigue, night sweats or weight loss to suggest a cancer diagnosis, the initial diagnosis of pyogenic liver abscess was pursued. The patient was admitted to the internal medicine service. Piperacillin/tazobactam and metronidazole were ordered, and interventional radiology (IR) was consulted for drainage of his liver abscesses the next day. The patient had no further fevers after drainage of his abscesses. Drainage Gram stain was negative, and cultures showed no growth during hospitalization. The infectious disease service was consulted and recommended downgrading his antibiotics from piperacillin/tazobactam to amoxicillin/clavulanate. On hospital day five, the patient tested positive for *Entamoeba histolytica* antibodies, which led to his final diagnosis of amoebic liver abscess. He was restarted on metronidazole, and paromomycin was initiated by the time of his hospital discharge. It was ultimately thought that the patient had been infected with *E. histolytica* on his prior military tours and had a dormant cyst that was reactivated when started on the infliximab infusions.

## RESIDENT DISCUSSION (Dr. Chu)

Liver abscesses are primarily classified as either pyogenic or amoebic depending on the cause. A pyogenic liver abscess is the most common intra-abdominal organ abscess.^4^ Most pyogenic liver abscesses are polymicrobial, although enteric Gram-negative bacilli and streptococci can commonly be identified in abscess specimens.^5^,^6^ The disease generally occurs in patients with predisposing factors such as diabetes mellitus, hepatobiliary disease, or current proton pump inhibitor use.^7^,^8^ Common clinical symptoms include fever, abdominal pain, nausea, vomiting, and malaise, although fever and abdominal pain are among the most frequently seen in patients.^9^

Amoebic liver abscesses are commonly caused by *E. histolytica*, which is a protozoan parasite that is predominantly found in developing regions. Over 100,000 annual deaths are attributed to *E. histolytica* infection.^10^ When diagnosed in patients living in more developed regions, those infected are likely to be migrants from endemic regions or travelers to areas such as Africa, Central and South America, Mexico, and South Asia, where they have had contact with fecal contaminated sources of food, water, and/or to a lesser extent, sexual transmission.^11^,^12^ *E. histolytica* exists in two forms: the cyst form, which is the infectious form that begins after ingestion; and the trophozoite form, which is the invasive form of the disease that can cause tissue inflammation and ultimately necrosis in intestinal sites or sites such as the liver (the most common extra-intestinal manifestation), brain, lung, or heart.^11^,^13^ Although most *E. histolytica* infections are asymptomatic, symptoms are similar to a pyogenic liver abscess. Symptoms may or may not also include diarrhea, weight loss, and bloody stools, depending on whether there is a concurrent intestinal infection.^11^

The imaging test of choice for any liver abscess comes down to ultrasound vs CT with IV contrast. Computed tomography is considered more sensitive with up to a 97% sensitivity.^14^ Subsequent work-up of a liver abscess seen on imaging usually entails needle aspiration by IR for both diagnostic and/or therapeutic purposes.^11^ If pyogenic, Gram stains will show leukocytes and bacteria. *E. histolytica*, on the other hand, may be diagnosed by stool microscopy, stool antigen testing, stool polymerase chain reaction, or serology. Because most cases of amoebic abscesses may occur without intestinal infection, there is a lower sensitivity to stool studies and, thus, serology is preferred.^5^,^11^

In addition to drainage, antibiotic treatment is also necessary. For pyogenic liver abscesses, antibiotic selection starts with broad-spectrum coverage with empiric *E. histolytica* coverage until the parasitic infection is ruled out. This is most typically done with piperacillin/tazobactam with metronidazole (to provide coverage for *E. histolytica*), or a third generation or later cephalosporin with metronidazole, and then tailored for coverage of aspirate growth cultures. The type of treatment needed to eliminate *E. histolytica* depends on the patient’s presentation. Asymptomatic patients are treated with an intraluminal agent, such as paromomycin, iodoquinol, or diloxanide furoate, to prevent disease progression and transmission. Symptomatic patients, however, are treated with an intraluminal agent after they have received systemic therapy with a tissue active agent such as metronidazole.

## FINAL DIAGNOSIS

*Entamoeba histolytica* liver abscess

**KEY TEACHING POINTS**

- Most *Entamoeba histolytica* infections are asymptomatic, but symptomatic patients may have recurrent fever and abdominal pain.
- Symptomatic patients require both an intraluminal agent, such as paromomycin, AND a tissue active agent, such as metronidazole, for elimination of the parasite.
- Undifferentiated fevers in the emergency department have a broad differential. Consider using the fever of unknown origin mnemonic I-MADE as a starting point.

## Figures and Tables

**Table:** Initial laboratory results of a 57-year-old male veteran with recurrent fevers.

Test name	Patient value	Reference range
Complete blood count		
White blood cells	12.6 K/mcL	4.5 – 11 K/mcL
Hemoglobin	14.2 g/dL	11.9 – 15.7 g/dL
Hematocrit	42.3%	35.0 – 45.0%
Platelets	285 K/mcL	153 – 367 K/mcL
Complete metabolic panel		
Sodium	135 mmol/L	136 – 145 mmol/L
Potassium	3.6 mmol/L	3.5 – 5.1 mmol/L
Chloride	101 mmol/L	98 – 107 mmol/L
Bicarbonate	19 mmol/L	21–30 mmol/L
Blood urea nitrogen	11 mg/dL	7 – 17 mg/dL
Creatinine	1.0 mg/dL	0.52 – 1.04 mg/dL
Glucose	105 mg/dL	70–100 mg/dL
Albumin	2.9 g/dL	3.2 – 4.6 g/dL
Total bilirubin	0.4 mg/dL	0.3 – 1.2 mg/dL
Aspartate aminotransferase	66 units/L	14 – 36 units/L
Alanine aminotransferase	54 units/L	0 – 34 units/L
Alkaline phosphatase	73 units/L	38 – 126 units/L
Anion gap	15 mmol/L	6–15 mmol/L
Coagulation		
Prothrombin Time	18.2 seconds	12.1–15.0 seconds
Partial thromboplastin time	33.4 seconds	25–38 seconds
International normalized ratio	1.6	0.8–1.1
Urinalysis		
Appearance	Clear	Clear
Color	Yellow	
Bilirubin	Negative	Negative
Ketones	Negative	Negative
Leukocyte esterase	Negative	Negative
Mucus	1+	Negative
Nitrite	Negative	Negative
pH	5.50	5.0–8.0
Protein	1+	Negative
Specific gravity	1.024	1.002–1.030
Blood	1+	Negative
Glucose	Negative	Negative
Urobilinogen	4.0	Negative
White blood cells	0–5 cells/hpf	0–5 cells/hpf
Red blood cells	0–5 cells/hpf	0–2 cells/hpf
Additional labs		
SARS-CoV-2	Negative	Negative
Lactate	0.9 mmol/L	0.5–2.2 mmol/L

*K*, thousands; *mcL*, microliter; *g*, grams; *dL*, deciliter; *mmol*, millimole; *L*, liter; *mg*, milligram; *dL*, deciliter; *hpf*, high powered field; *SARS-CoV-2*, severe acute respiratory syndrome coronavirus 2.
